# Similarities and differences between Alzheimer's disease and schizophrenia: drug target Mendelian randomization and transcriptome analysis

**DOI:** 10.7150/ijms.119636

**Published:** 2026-01-01

**Authors:** Yuping Zhang, Ju Shao, Bibo Liang, Yihong He, Zhixin Wen, Bo Cai, Ligang Jie

**Affiliations:** 1 Department of Rheumatology and Clinical Immunology, Zhujiang Hospital, Southern Medical University, Guangzhou, China.; 2 Xinjiang medical university, Xinjiang, China.

## Abstract

**Background:** Alzheimer's disease (AD) and schizophrenia (SZ) are two complex neuropsychiatric disorders characterized by significant cognitive impairment. The relationship between them is complex and multifaceted. Understanding these similarities and differences is crucial for identifying common therapeutic targets.

**Methods:** Genetic data on plasma proteome were obtained from Icelandic datasets. Genetic variants associated with circulating inflammatory proteins, rheumatoid arthritis (RA) and AD and SZ, were sourced from large GWAS datasets. Mendelian randomization analyses were performed, and transcriptome analysis were applied to confirm the finding.

**Results:** We observed an association between the genetic loci of the AD and SZ traits (p < 0.05) by Linkage Disequilibrium Score Regression. Furthermore, our analysis indicates that RA has a protective effect against AD (OR: 0.36, p = 0.005) while potentially increasing the risk for SZ (OR: 2.49, p = 2.06 × 10⁻⁷). Among the 1,729 proteins analyzed, 16 proteins exhibited significant inverse causal relationships with AD and positive causal relationships with SZ. Notably, DECR2 emerged as a potential therapeutic target, showing opposing effects in AD and SZ as revealed by SMR analysis and colocalization.

**Conclusions:** This study identifies causal proteins linked to AD and SZ, enhancing the understanding of their molecular etiology and supporting targeted therapeutics development.

## Introduction

Rheumatoid arthritis (RA) is a chronic autoimmune disorder that primarily targets the joints but also has significant systemic implications, including potential impacts on cognitive function 1, 2. Alzheimer's disease (AD) and schizophrenia are notable for their severe cognitive impacts. Interestingly, emerging research suggests that the relationship between RA and these cognitive disorders is complex and multifaceted 2,3,4,5,6.

Alzheimer's disease (AD) and schizophrenia (SZ) are two complex neuropsychiatric disorders characterized by significant cognitive impairment 7, 8. While AD primarily manifests as progressive memory loss and cognitive decline in older adults, SZ is characterized by psychosis, delusions, and disorganized thinking, typically beginning in early adulthood. Despite their distinct clinical presentations, increasing evidence suggests that AD and SZ share certain neurobiological mechanisms, including altered neurotransmitter pathways, synaptic dysfunction, and genetic overlaps. Understanding these similarities is crucial for identifying common therapeutic targets 9.

However, there are also critical differences between the two diseases, particularly in their molecular and pathological underpinnings. AD is marked by amyloid-beta plaques and tau tangles 10, while SZ involves disruptions in neurodevelopmental processes and dopamine regulation. Exploring these differences is essential to better understand the unique aspects of each disorder and to develop targeted interventions that address their distinct pathological mechanisms.

Interestingly, the differential relationships of both AD and SZ with the common autoimmune disease rheumatoid arthritis (RA) offer a valuable perspective for dissecting the shared and unique mechanisms of these two disorders. While RA has been associated with a protective effect against AD, it appears to exacerbate the risk of SZ, suggesting an important interplay between inflammation, immunity, and cognitive dysfunction. Studying these contrasting relationships provides an opportunity to uncover mechanisms that distinguish AD from SZ and to identify potential biomarkers and therapeutic targets.

Given the intertwined yet divergent nature of AD and SZ, studying their shared and unique features, including their differential relationships with RA, provides an opportunity to uncover biomarkers and therapeutic targets that are more specific and effective. This study leverages proteome-wide Mendelian randomization (MR) and transcriptome analysis to investigate the causal relationships between plasma proteins and these two disorders. By examining proteins with opposing roles in AD and SZ, we aim to identify novel therapeutic targets that could address the unique and overlapping aspects of these cognitive disorders, ultimately contributing to a deeper understanding of their molecular etiology and advancing precision medicine approaches.

## Methods

### Data sources for plasma proteins

Plasma pQTL data were obtained from a large-scale integration study by Ferkingstad et al. (11), which provided pQTLs for 4,907 plasma proteins from 35,559 Icelanders. To identify cis-pQTLs, we applied the following selection criteria:** (1)** the pQTLs had genome-wide significant associations (p < 5 × 10⁻⁸); **(2)** the pQTLs were located outside the major histocompatibility complex (MHC) region; (3) pQTLs were cis-acting, defined as pQTLs within a 1000 kb window around the corresponding protein-coding sequences; (4) independence was ensured through linkage disequilibrium (LD) clumping (r² < 0.001); (5) the pQTLs were not weak instrumental variables (IVs), with F-statistics greater than 10; and (6) palindromic single nucleotide polymorphisms (SNPs) and SNPs with missing data were excluded. Ultimately, 6,238 cis-acting SNPs for 1,729 proteins were included in the analysis (Supplementary Table S1).

### Data sources for rheumatoid arthritis, Alzheimer's disease, and schizophrenia

Data on the associations of SNPs with rheumatoid arthritis were obtained from the GWAS meta-analysis conducted by Dönertaş, H.M.et al 11, which included 5427 RA cases and 479,171 controls. Data for Alzheimer's disease were sourced from the GWAS meta-analysis conducted by Lambert J.C.et al 12, which included 17,008 AD cases and 37154 controls. Schizophrenia data were obtained from the Schizophrenia Working Group of the Psychiatric Genomics Consortium, including 33,640 cases and 43,456 controls 13. There were no sample overlaps between the outcome datasets. The validation and extension sets used in this study are detailed in Table 1. These datasets were employed to confirm and expand upon the findings from the primary analysis, ensuring the robustness and generalizability of the identified associations between plasma proteins and their potential roles as therapeutic targets in Alzheimer's disease (AD) and schizophrenia (SZ).

### Linkage disequilibrium score regression

Linkage Disequilibrium Score Regression (LDSC) was performed to first estimate the heritability of two traits, Alzheimer's disease (AD) and schizophrenia (SZ), and subsequently calculate the genetic correlation between them. Significant genetic correlations, identified after applying Bonferroni correction (p < 0.05), were visualized using a heatmap.

### Data sources for circulating inflammatory proteins

Genetic variants associated with circulating inflammatory proteins were identified from a comprehensive genome-wide meta-analysis, which analyzed 91 plasma proteins in a sample of 14,824 individuals of European descent, spanning 11 distinct cohorts.12 This study utilized the Olink Target-96. The data on these 91 plasma inflammatory proteins, including the pQTL findings, are accessible in the EBI GWAS Catalog (accession numbers GCST90274758 to GCST90274848).

### MR analysis

MR analyses were performed using TwoSampleMR and MendelianRandomization packages in R software (4.4.2). All MR analyses fulfilled three core assumptions 14. First, genetic variants must be vigorously associated with the exposure. Second, genetic variants must not be associated with confounders of the associations between instruments of each exposure and each outcome. Third, the effects of genetic variants on each outcome must go through each exposure. We used the random-effect IVW method as the main analysis. We utilized a two-sample MR approach to estimate the causal effects of plasma proteins on the risk of RA, AD, and SZ. For each protein, the Wald ratio method was used when a single SNP was available, and the inverse-variance weighted (IVW) method was employed when multiple SNPs were available.

### Summary-data-based Mendelian randomization

This approach uses expression quantitative trait loci (eQTLs) as instruments to assess the association between gene expression levels and the outcomes of interest, leveraging summary-level data from genome-wide association studies (GWAS) and eQTL studies 15.

### Colocalization analysis

We performed colocalization analysis to test whether the identified associations of proteins with RA, AD, and SCZ were driven by linkage disequilibrium. The analysis was based on a Bayesian model that assesses the support for five exclusive hypotheses: 1) no association with either trait; 2) association with trait 1 only; 3) association with trait 2 only; 4) both traits are associated, but distinct causal variants were for two traits; and 5) both traits are associated, and the same causal variant is shared. A posterior probability is provided for each hypothesis testing (H0, H1, H2, H3, and H4). In this analysis, we used the setting with prior probabilities for the SNP being associated with trait 1 only (p1) at 1 x 10^-4, the probability for the SNP being associated with trait 2 only (p2) at 1 x 10^-4, and the probability for the SNP being associated with both traits (p12) at 1 x 10^-5. Two signals were considered to have strong support for colocalization if the posterior probability for shared causal variants (PP.H4) was ≥0.8. Medium colocalization indication was defined as 0.5 < PP.H4 < 0.8. The analysis was performed using the coloc package in R software (4.4.1). Given that the traditional colocalization approach cannot detect the scenario where the exposure and outcome traits share more than one causal hit, we utilized the SuSiE (Sum of Single Effects) colocalization method by integrating proteomics GWAS summary statistics and genetic correlation matrix reference panels based on individuals of European ancestry from the 1000 Genomes Project Phase 3 to identify multiple causal variants.

### KEGG and GO pathway analysis

KEGG is an open and widely used database integrating information on genomes, biological pathways, diseases, and drugs. KEGG pathway analysis was performed to mine pathways enriched in the list of significant proteins. To account for multiple testing, the FDR corrected P-value on the pathway less than 0.05 was significant. GO (Gene Ontology) analysis is to determine BP (biological process), CC (cellular component), and MF (molecular function) term enrichment.

### Transcriptome analysis

#### Dataset download and identiffcation of DEGs

We used “Alzheimer's disease” or “schizophrenia” as keywords to search for related datasets in GEO database (https://www.ncbi.nlm.nih.gov/geo/). The datasets for homo sapiens microarray analysis of AD (GSE5281) and SZ (GSE21935) were selected. Differential analysis was performed by microarray data linear model (limma) software package (version 3.40.6). The results of DEGs are presented by volcano map and heatmap.

## Results

**Figure 1:**
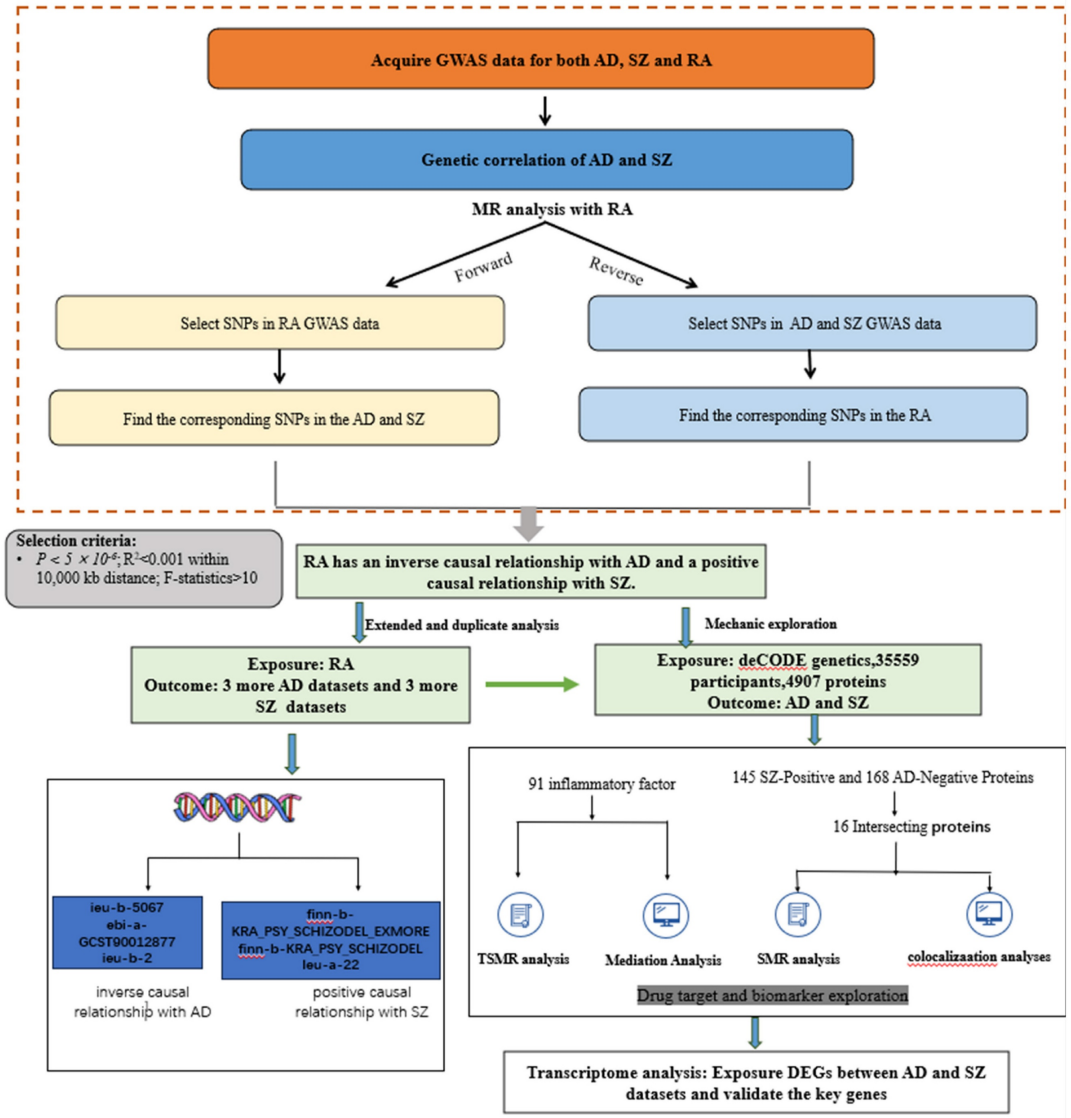
** An overview of the study design**.

An overview of the study design is shown in **Figure 1**. All analyses were based on summary-level data listed in **Table 1**. We conducted bidirectional Mendelian randomization analyses between rheumatoid arthritis (RA) and both Alzheimer's disease (AD) and schizophrenia (SZ). Based on these results, we further explored the underlying mechanisms by employing proteome-wide Mendelian randomization analysis.

### Genetic correlation of AD and SZ

**Figure 2:**
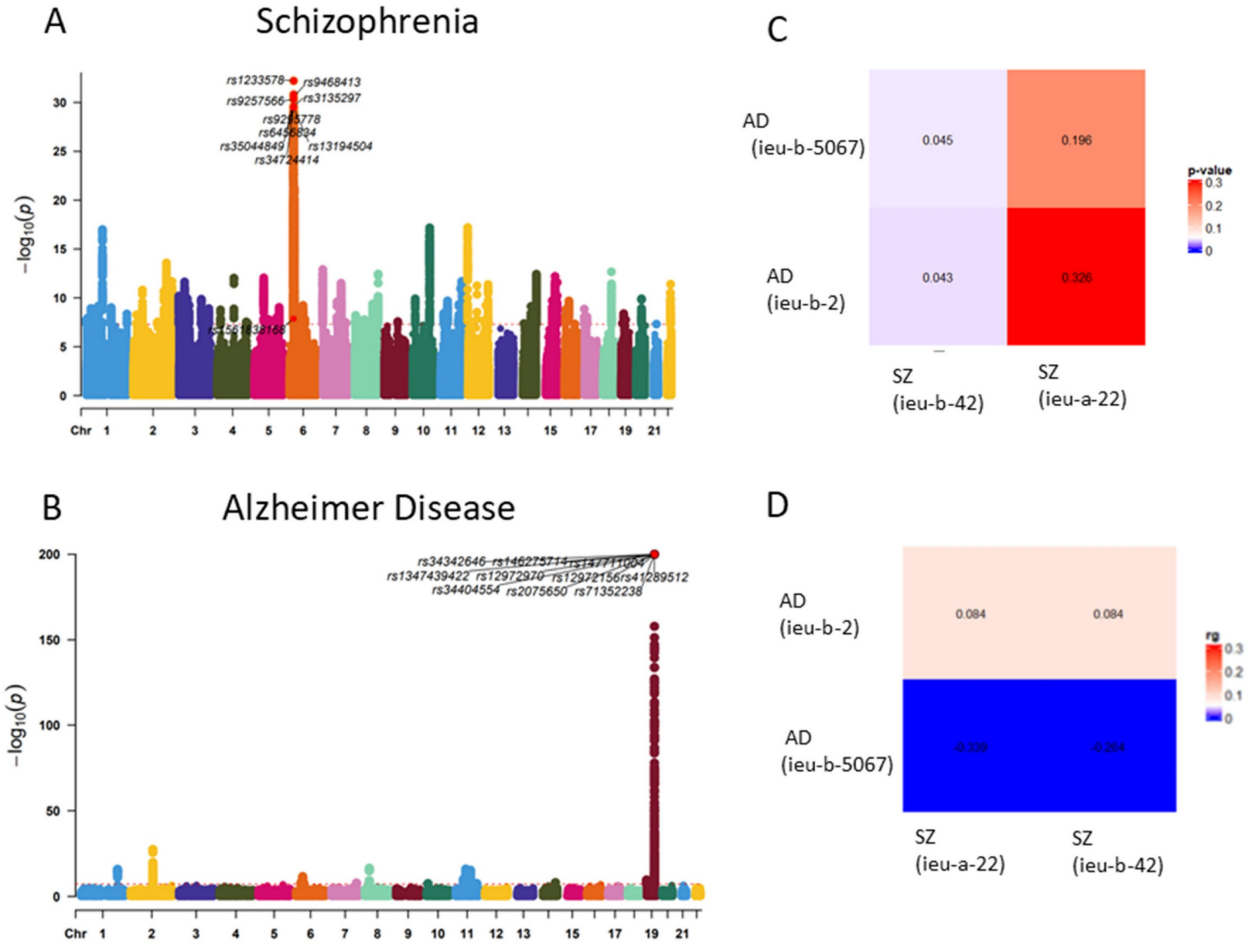
** Genetic correlation of OA and RA. (A)** Manhattan plot of Schizophrenia SNP loci. **(B)** Manhattan plot of Alzheimer Disease SNP loci. **(C)** Genetic correlation analysis of Schizophrenia and Alzheimer Disease (LDSC) (rg p value). **(D)** Genetic correlation analysis of Schizophrenia and Alzheimer Disease (LDSC rg)

GWAS analysis of Alzheimer's disease (AD) and schizophrenia (SZ) was conducted using data from the IEU database to investigate potential shared genetic factors. By comparing the datasets of AD and SZ with multiple GWAS analyses, we observed an association between the genetic loci of the two traits (p < 0.05). This suggests a potential genetic correlation between AD and SZ (**Figure 2**).

### Genetic GWAS MR analysis: genetic casual effect of rheumatoid arthritis on Alzheimer's disease and schizophrenia

**Figure 3:**
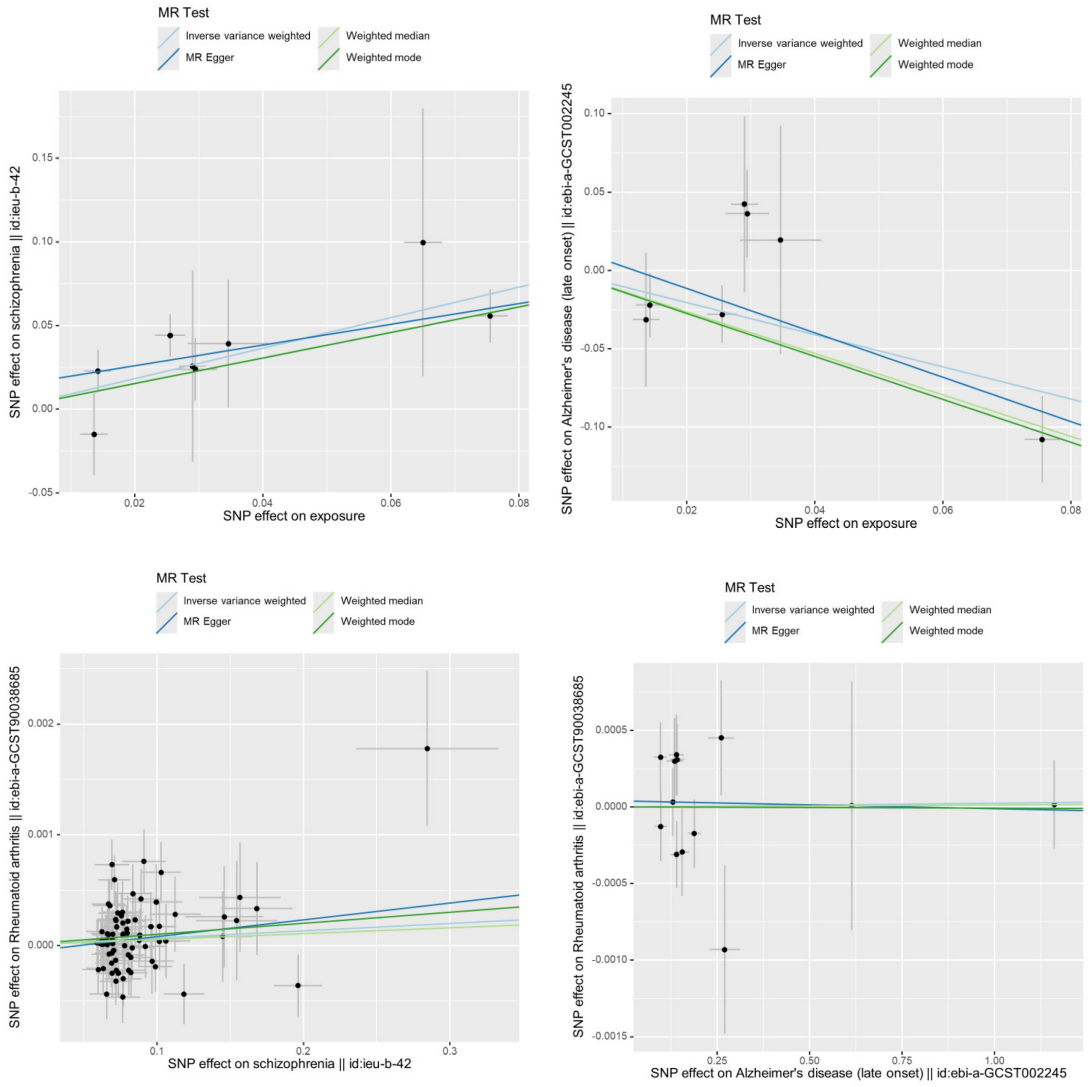
** Scatter plot of bidirectional two-sample MR analysis. (A)** Genetic casual effect of Rheumatoid Arthritis on schizophrenia; **(B)** Genetic casual effect of Rheumatoid Arthritis on Alzheimer's disease. **(C D)** Reverse mendelian randomization

The genetic instruments for RA, AD and SZ were robust, with F-statistics exceeding the threshold of 10, indicating strong instrument strength. Key results are summarized as follows:

**Alzheimer's disease:** The GWAS analysis demonstrated a significant association between RA and decreased risk of Alzheimer's disease (OR: 0.36, 95% CI: 0.17-0.74, p = 0.005) (**Figure 3, Table 2**) The findings indicated a protective effect of RA against AD. The reverse Mendelian randomization analysis yielded negative results.

**Schizophrenia:** The GWAS analysis demonstrated a significant association between RA and increased risk of Schizophrenia (OR: 2.49, 95% CI: 1.76-3.51, p =2.06*10^-7^) (**Figure 3, Table 2**) The findings indicated a promoting effect of RA against SZ. The reverse Mendelian randomization analysis yielded negative results.

#### Circulating inflammatory protein GWAS analysis and mediation analysis

The Mendelian randomization (MR) analysis focused on Circulating Inflammatory Proteins and their mediation of the relationship between RA and Alzheimer's disease and schizophrenia.

**Rheumatoid arthritis**: Six inflammatory factors, including Neurturin, Interleukin-6, Programmed cell death 1 ligand 1, CUB domain-containing protein 1, C-C motif chemokine 4, and CD40L receptor, are significantly associated with RA (Supplementary Table S2).

**Alzheimer**: Higher levels of C-C motif chemokine 20 and interferon gamma were associated with an increased risk of Alzheimer's disease (AD), while higher levels of monocyte chemoattractant protein-1 and C-X-C motif chemokine 9 were linked to a reduced risk of AD (Supplementary Table S3).

**Schizophrenia**: Higher levels of several inflammatory factors, including interleukin-6 and C-C motif chemokine 28, were associated with an increased risk of schizophrenia, while higher levels of CD40L receptor and other factors were linked to a reduced risk. The mediation analysis did not reveal any positive results (Supplementary Table S4).

#### Causal effects of plasma proteins on schizophrenia and Alzheimer's disease (AD)

By setting a significance threshold of p1=5e-8 and a cis-regulatory window of 1000kb, and by removing plasma proteins lacking genetic instruments, the MR analysis included cis-genetic instruments for 1,729 proteins.

In our comprehensive proteome-wide Mendelian randomization (MR) analysis, we employed cis-genetic instruments as exposure SNPs and analyzed several datasets for Alzheimer's disease (AD) and schizophrenia (SZ) as outcomes. All datasets pertaining to Alzheimer's disease (AD) and schizophrenia (SZ) are summarized in** Table 1**. In the two-sample Mendelian randomization (MR) analyses involving rheumatoid arthritis (RA) and datasets for Alzheimer's disease (AD) and schizophrenia (SZ), the results consistently demonstrated similar findings across all datasets (**Supplementary Figure S1**).

### Overlap of SZ-positive and AD-negative proteins

**Figure 4:**
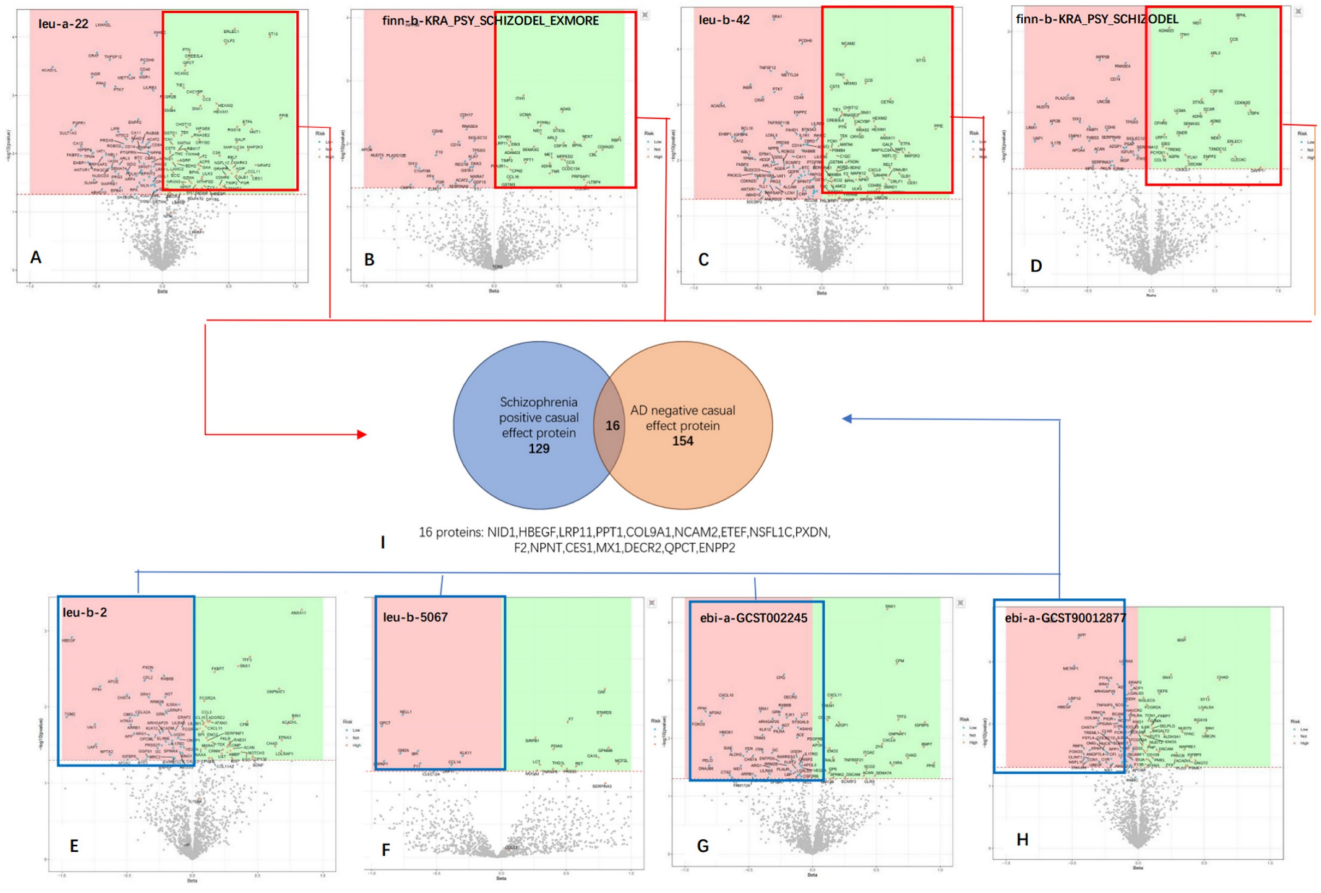
** Illustration of the screening process for the Overlap of SZ-Positive and AD-Negative Proteins. (A B C D)** The volcano plot shows the results of proteomic analyses conducted on four different SZ GWAS datasets, with a combined total of 129 proteins identified as positively associated across all datasets.** (E F G H)** The volcano plot displays the results of proteomic analyses performed on four different AD GWAS datasets, revealing a total of 154 proteins identified as negatively associated across all datasets.** I** Overlap of SZ-Positive and AD-Negative Proteins.

In the proteomics Mendelian randomization (MR) analysis, all proteins that were positively associated with schizophrenia (SZ) and negatively associated with Alzheimer's disease (AD) and deemed statistically significant were combined into a unified set for further investigation (**Figure 4**). We identified 145 proteins that were positively associated with SZ and 168 proteins negatively associated with AD. The intersection analysis revealed 16 proteins that have statistically significant inverse causal relationships with Alzheimer's disease (AD) and positive causal relationships with schizophrenia (SZ). These proteins are as follows: NID1, HBEGF, LRP11, PPT1, COL9A1, NCAM2, ETEF, NSFL1C, PXDN, F2, NPNT, CES1, MX1, DECR2, QPCT and ENPP2.

To gain insights into the biological functions and pathways associated with the intersecting proteins identified from the MR analysis, we performed Kyoto Encyclopedia of Genes and Genomes (KEGG) pathway and Gene Ontology (GO) enrichment analyses.

### KEGG pathway analysis

Several pathways were identified with uncorrected p-values < 0.05. These pathways, though not statistically significant after correction, include: 1. Coronavirus disease - COVID-19; 2. ECM-receptor interaction; 3. Virion - Ebolavirus, Lyssavirus and Morbillivirus, 4. Fatty acid elongation Cytoskeleton in muscle cells.

### GO enrichment analysis

**Figure 5:**
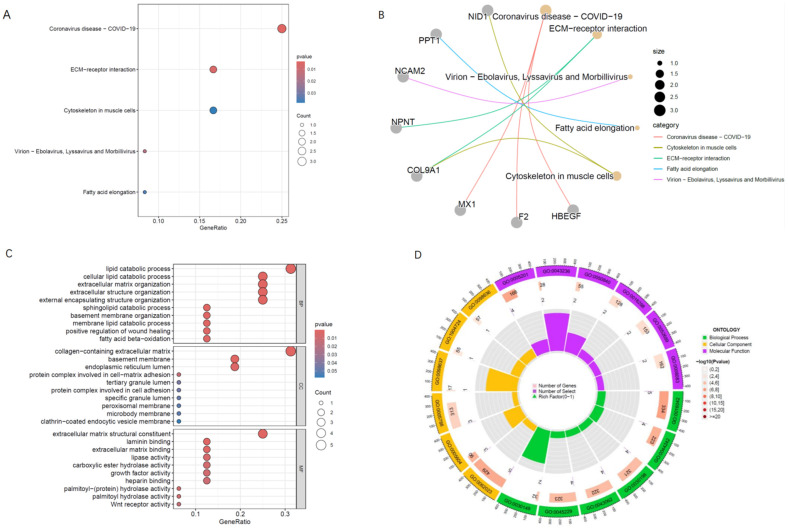
** (A, B)** The bubble chart and cnetplot represent the KEGG analysis of the 16 proteins that overlap between SZ-positive and AD-negative associations** (C, D)** GO The bubble chart and circular plot represent the KEGG analysis of the 16 proteins. Among the eight statistically significant GO pathways identified, four are related to lipid metabolism, and four are associated with extracellular matrix organization.

In the Gene Ontology (GO) enrichment analysis of the 16 intersecting proteins, several pathways were found to be statistically significant even after correction for multiple testing. Top three pathways with statistically significant adjusted p-values were: Lipid Catabolic Process; Cellular Lipid Catabolic Process; Extracellular Matrix Organization. Among the eight statistically significant GO pathways identified, four are related to lipid metabolism, and four are associated with extracellular matrix organization **(Figure 5).**

### Exploring potential therapeutic targets and biomarkers

The analysis of proteins that simultaneously exhibit protective effects against Alzheimer's disease (AD) and promote schizophrenia (SZ) provides a unique opportunity to explore specific therapeutic targets and biomarkers for these conditions.

### SMR and colocalization analysis

To identify potential therapeutic targets among the proteins showing a positive causal relationship with schizophrenia (SZ) and a negative causal relationship with Alzheimer's disease (AD), we applied the summary-data-based Mendelian randomization (SMR) method.

**Figure 6:**
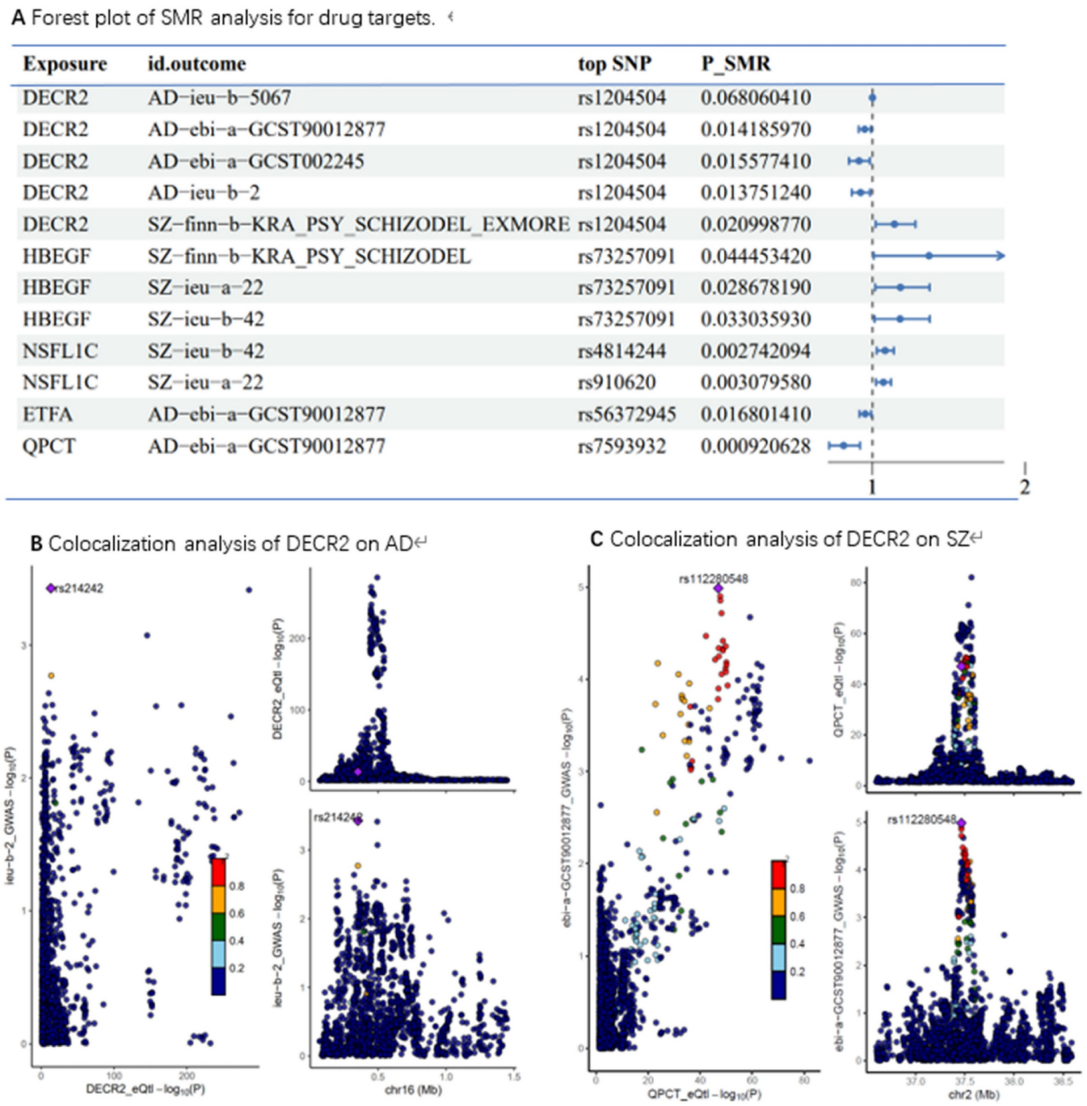
** (A)** Forest plot of SMR analysis for drug targets;** (B)** Colocalization analysis of DECR2 on AD, PP.H4=0.87; **(C)** Colocalization analysis of DECR2 on SZ, PP.H4= 0.41.

The results of the analysis are presented in **Figure 6**. The proteins highlighted in the figure represent promising candidates for further investigation as drug targets including: DECR2, HBEGF, NSFL1C, ETFA and QPCT. Notably, the SMR results identified DECR2 can serve as a protective drug target for AD and a promotive drug target for SZ.

To further validate our findings, we conducted colocalization analysis to determine whether the identified proteins shared a causal variant with AD and SZ. The colocalization analysis provided strong support for shared causal variants for the majority of the significant proteins identified in the MR analysis. The posterior probability (PP) of hypothesis 4 (PP.H4) being ≥0.8 was considered strong evidence of colocalization. For AD, DECR2 demonstrated PP.H4 values of 0.87 indicating strong colocalization. Similarly, for SZ, DECR2 showed PP.H4 values of 0.41.

### Identiffcation of common and differential genes between AD and SZ and validate the key genes

To further investigate the similarities and differences between Alzheimer's disease (AD) and schizophrenia (SZ), we used transcriptome data to identify common and differential genes associated with these two traits. The dataset GSE5281 contained 13 normal control samples (EC), 10 AD samples. The dataset GSE 21935 contained 23 normal control samples and 10 SZ samples. 7363 DEGs were identified between AD and normal controls, and 9 DEGs were found to overlap between AD and SZ. 905 DEGs were identified between SZ and normal controls, and 9 DEGs were found to overlap between AD and SZ.

**Figure 7:**
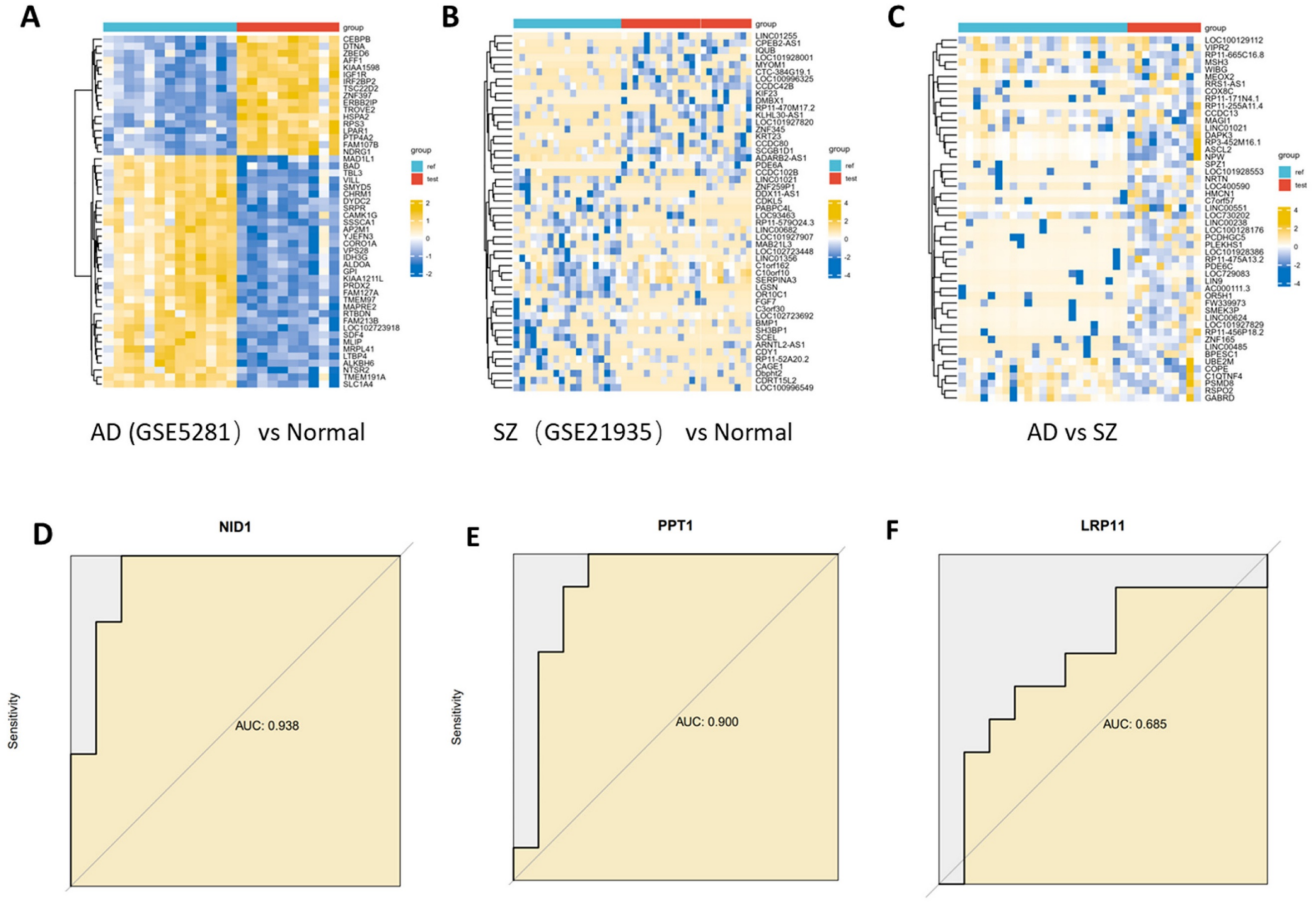
Differentially expressed genes identiffcation. **(A)** Heatmap of top DEGs between AD and normal control. **(B)** Heatmap of top DEGs between SZ and normal control. **(C)** Heatmap of top DEGs between AD and SZ. **(D, E, F)** Key gene were assessed using AUC (area under the curve) analysis. NID1 showed an AUC of 0.938, PPT1 had an AUC of 0.900.

To validate the key proteins identified from the analysis above, their RNA expression levels were assessed using AUC (area under the curve) analysis. NID1 showed an AUC of 0.938, suggesting high discriminatory power for AD compared to normal controls. Similarly, PPT1 had an AUC of 0.900 for distinguishing AD from normal controls **(Figure 7)**.

## Discussion

Alzheimer's disease (AD) and schizophrenia (SZ) are both cognitive disorders, yet they exhibit significant differences in their clinical manifestations. Recent studies have identified similarities in the cognitive impairment mechanisms between Alzheimer's disease (AD) and schizophrenia (SZ), such as α7 Nicotinic receptor 16, 17. However, our research focuses more on the differences between these two conditions, as these differences may better capture the fundamental characteristics of each disease. Understanding these distinctions is particularly important when exploring protective drug targets, as it can lead to the discovery of significant insights that are often missed in conventional single-disease versus control group analyses.

Rheumatoid arthritis (RA) patients typically share a specific genetic background 18. In mendelian randomization analyses, the RA GWAS dataset exhibits a positive causal relationship with schizophrenia (SZ) and a negative causal relationship with Alzheimer's disease (AD). This intriguing contrast prompted us to conduct further research to explore the underlying mechanisms and implications of these divergent genetic associations. GWAS data can be complex, with numerous confounding factors such as population background. The innovation in this study lies in its approach to filtering relevant AD and SZ datasets by leveraging their relationship with rheumatoid arthritis (RA). By focusing on this specific genetic link, the study strategically combines and analyzes only the proteins that meet the criteria, thereby reducing noise and enhancing the accuracy of the findings.

Evidence suggests that RA may confer a protective effect against AD, potentially due to the anti-inflammatory properties of treatments commonly used for RA, such as disease-modifying antirheumatic drugs (DMARDs). A study conducted using the Taiwan Longitudinal Health Insurance Database found that individuals with RA had a significantly lower prevalence of AD compared to those without RA, indicating a possible protective effect. 2 Conversely, the association between RA and SZ appears to be more complex. While some studies suggest a reduced risk of RA in individuals with SZ 19, others have found no significant correlation or have reported an increased risk of SZ in individuals with a family history of RA 20. In this study, we utilized recently published large-scale GWAS data to investigate the relationship between rheumatoid arthritis (RA) 11, Alzheimer's disease (AD) 12, 21, 22, and schizophrenia (SZ) 13 through Mendelian randomization analysis. Interestingly, our study revealed that RA is protective against AD while promoting SZ.

To investigate the shared key factors that protect against AD and promote SZ, we further conducted a proteomics Mendelian randomization analysis, with a strategic focus on analyzing the Intersection of SZ-Positive and AD-Negative Proteins. The deCODE protein data used in our analysis is a significant resource in genetic and proteomic research, particularly known for its comprehensive collection of human genetic data 23. Previous studies have explored the plasma proteomics and both AD 24-26 and SZ 27, 28. However, this study is the first to investigate potential drug target by utilizing the approach of identifying differential proteins between the two diseases 29.

Among the eight statistically significant GO pathways identified, four are related to lipid metabolism, and four are associated with extracellular matrix organization. This is partly consistent with previous research findings 30. There has long been evidence supporting the association between lipid metabolism and neurodegenerative processes 22, 31. This study employed a dual-disease proteomic Mendelian randomization approach to pinpoint specific lipid metabolism pathways and targets.

Our analysis identified DECR2 as a dual-purpose biomarker, serving as a protective target for AD and a promotive target for SZ. DECR2 (2,4-Dienoyl-CoA Reductase 2) is an enzyme involved in fatty acid metabolism, specifically in the breakdown of unsaturated fatty acids within the mitochondria. This protein plays a crucial role in the β-oxidation pathway, which is essential for cellular energy production 32 Our study identify DECR2 as a potential therapeutic target for Alzheimer's disease (AD). Insights drawn from an extensive review of the literature indicate that DECR2's involvement in modulating lipid metabolism and mitochondrial functionality may underlie its therapeutic potential 33, 34. Alterations in astrocytic lipid metabolism can lead to neuroinflammation and oxidative stress, both of which are implicated in the progression of AD 35-37. Recent studies 38, 39 further suggest that dysregulated fatty acid oxidation may impair astrocytic mitochondrial function, activate neuro-inflammatory signaling, and disrupt synaptic plasticity, thereby accelerating neurodegeneration. These mechanistic insights provide additional biological plausibility for the protective effect of DECR2 in AD.

The role of DECR2 in SZ is less clear, but there is growing evidence that metabolic disturbances, including lipid metabolism, may be involved in the pathophysiology of SZ 40, 41. Changes in fatty acid oxidation processes could affect neurotransmitter synthesis and synaptic function, potentially contributing to the symptoms of SZ 42. It is plausible that DECR2 dysregulation exacerbates neuroinflammatory signaling in SZ, contrasting with its protective role in AD, though further research is needed to elucidate these pathways.

In addition to identifying DECR2 as a potential therapeutic target, our study also uncovered several other promising drug targets, such as NSFL1C, HBEGF for SZ and ETFA and QPCT for AD**.** In the context of SZ, HBEGF and its related signaling pathways, particularly through the ErbB family of receptors, are thought to be involved in neurodevelopmental and synaptic processes 43. Abnormalities in these pathways may contribute to the synaptic dysfunction and cognitive deficits characteristic of SZ. Research indicates that alterations in HB-EGF expression influence neuroinflammatory responses and synaptic signaling, thereby playing a role in the pathophysiology of SZ 44. The roles of NSFL1C and ETFA 45 in neurological diseases have been reported infrequently. There have been several reports suggesting a potential role of QPCT in Alzheimer's disease (AD) 46, 47.

While these protein-level findings highlight potential therapeutic avenues, we also investigated whether circulating inflammatory proteins might mediate the observed associations between RA and neuropsychiatric disorders. However, the lack of significant mediation results may be due to weak causal estimates of RA on inflammatory proteins and limited instrument strength. Nevertheless, extensive experimental and clinical evidence supports the role of inflammation in both AD and SZ 48, 49. Thus, our null findings should be viewed as reflecting methodological constraints rather than excluding the biological relevance of inflammatory pathways.

This study also has several limitations. First, the identification of drug target proteins, requires further validation. These proteins have not been widely studied in the context of neurological and psychiatric disorders, and their exact roles in disease mechanisms remain to be elucidated. Second, the transcriptomic validation is underpowered due to the small sample sizes of the GEO datasets (GSE5281 and GSE21935), each containing fewer than 15 AD and 15 SZ samples. This limitation reduces the robustness of the reported expression changes for key genes such as NID1 and PPT1, underscoring the need for validation in larger transcriptomic and proteomic cohorts. The limited sample size may also inflate the uncertainty of the estimated effects, resulting in wider confidence intervals. Therefore, replication in larger eQTL and pQTL consortia, such as eQTLGen and UKB-PPP, is warranted to confirm these associations before any therapeutic validation. Additionally, the study's reliance on existing genetic and proteomic databases may limit the comprehensiveness of the findings, as not all potential target proteins and pathways are fully represented in these datasets. Finally, the study's findings are primarily based on bioinformatic analyses, which need to be complemented by experimental and clinical research to confirm the therapeutic relevance of these targets.

In summary, this dual-disease proteomic Mendelian randomization and colocalization analysis identified several lipid metabolism pathways and proteins potentially involved in the pathogenesis of both Alzheimer's disease (AD) and schizophrenia (SZ). Among these proteins, a number exhibited distinct roles in the pathophysiology of each condition, highlighting the importance of understanding the divergent mechanisms underlying these cognitive disorders. The study not only confirmed DECR2 as a potential protective target but also uncovered additional therapeutic targets that could be crucial in developing more tailored treatment strategies. These findings emphasize the value of exploring disease-specific pathways to identify novel drug targets, particularly those that might offer protective effects against one condition while addressing the risks of another. Further research into these targets could lead to significant advancements in the treatment of AD and SZ, offering new hope for more effective and personalized therapies.

## Supplementary Material

Supplementary figures and tables.

## Figures and Tables

**Table 1:** Diseases and traits included in this study.

Disease or trait	Cases	Controls	Total	Reference (PMID)	Dataset classification
Rheumatoid arthritis	5,427	479,171	484,598	33959723	Analysis Set
Schizophrenia	33,640	43,456	77,096	25056061	Analysis Set
Alzheimer's disease	17,008	37,154	55,134	24162737	Analysis Set
Plasma protein	35,559	-		34857953	deCODE Analysis Set
Schizophrenia	10,091	166,584	176675	FinnGen R9	Validation and Extension Set
Schizophrenia	10,091	208,701	218792	FinnGen R9	Validation and Extension Set
Schizophrenia	35,476	46,839	82,315	25056061	Validation and Extension Set
Alzheimer's disease	-	-	472,868	33589840	Validation and Extension Set
Alzheimer's disease	954	487,331	488,285	NA	Validation and Extension Set
Alzheimer's disease	21,982	41,944	63,926	30820047	Validation and Extension Set

**Table 2:**
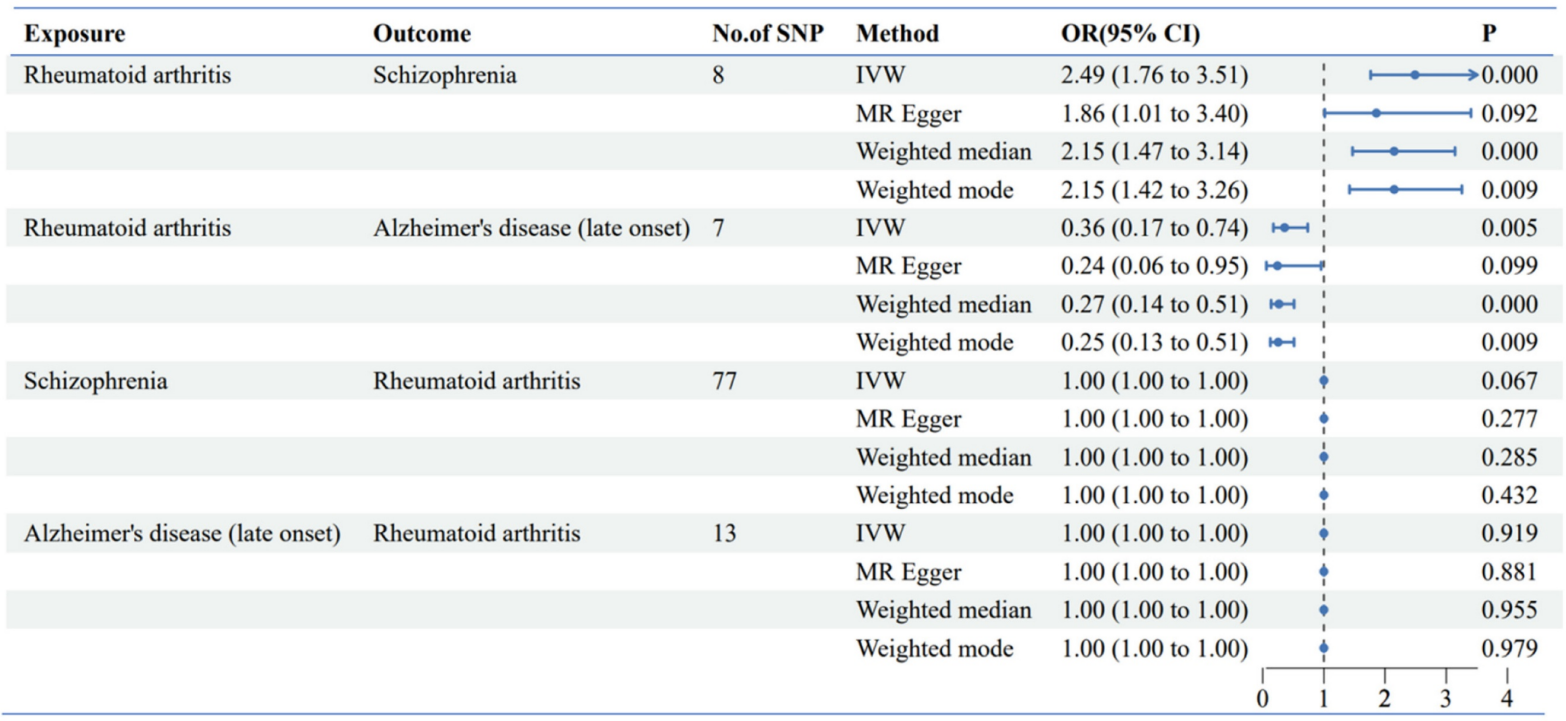
Genetic casual effect of Rheumatoid Arthritis on Alzheimer's disease and schizophrenia by bidirectional two-sample MR analysis.
